# Supplementary material for: p16Ink4a and p21Cip1/Waf1 promote tumour growth by enhancing myeloid-derived suppressor cells chemotaxis
Source: Nat Commun. 2017 Dec 12;8:2050. doi: 10.1038/s41467-017-02281-x (PMC5727112; doi:10.1038/s41467-017-02281-x)
Supplement: Supplementary file 1 — Supplementary Information [file 41467_2017_2281_MOESM1_ESM.pdf]

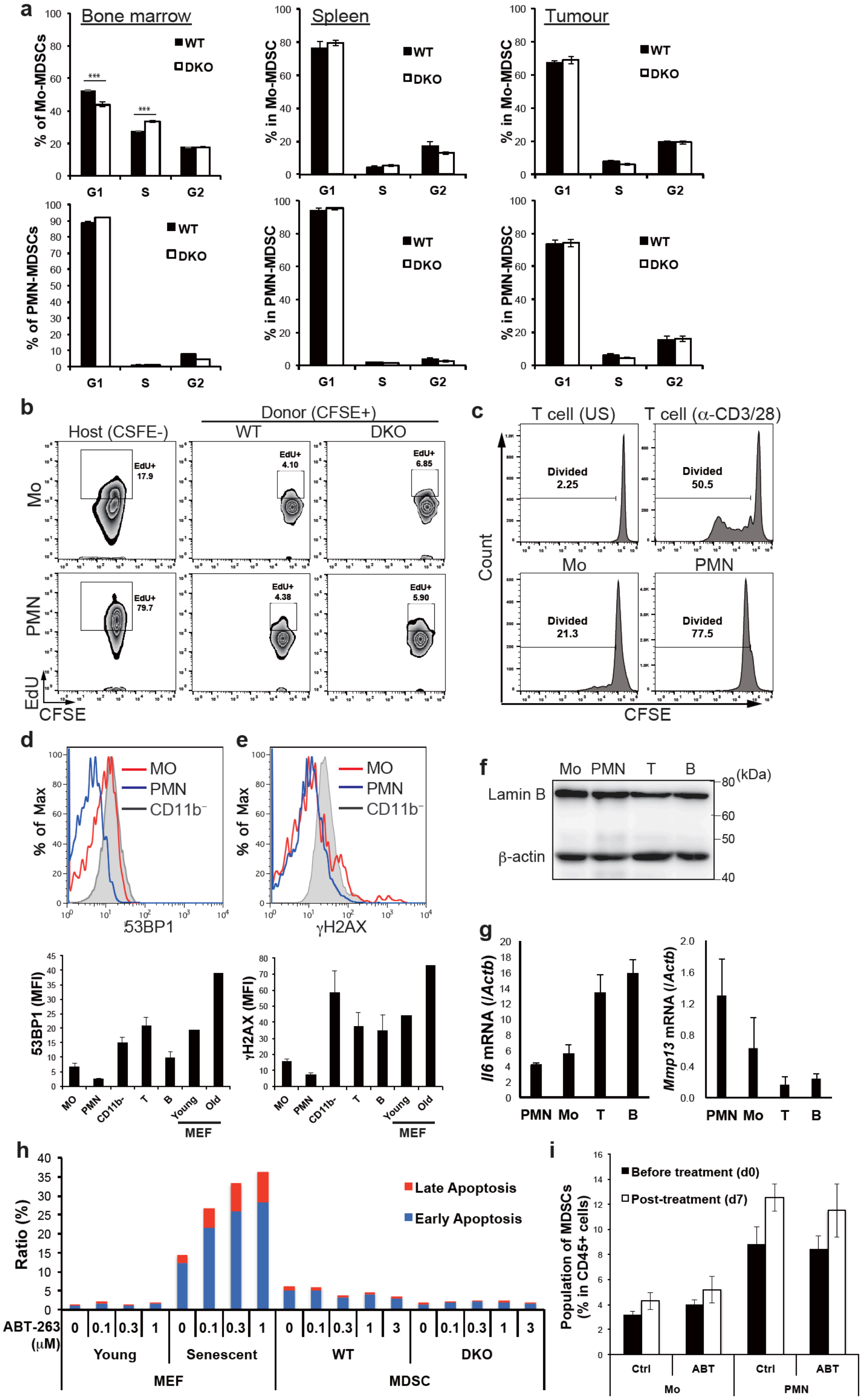
a
Bone marrow
Spleen
Tumour
WT
DKO
% of Mo-MDSCs
% in Mo-MDSC
% of PMN-MDSCs
% in PMN-MDSC
G1
S
G2
b
Donor (CFSE+)
Host (CFSE-)
WT
DKO
Mo
PMN
EdU+ 17.9
EdU+ 4.10
EdU+ 6.85
EdU+ 79.7
EdU+ 4.38
EdU+ 5.90
EdU
CFSE
c
T cell (US)
T cell (α-CD3/28)
Divided 2.25
Divided 50.5
Mo
PMN
Divided 21.3
Divided 77.5
Count
CFSE
d
e
MO
PMN
CD11b⁻
% of Max
53BP1
γH2AX
53BP1 (MFI)
γH2AX (MFI)
CD11b-
T
B
Young
Old
MEF
f
Mo PMN T B
(kDa)
Lamin B
β-actin
80
60
50
40
g
Il6 mRNA (/Actb)
Mmp13 mRNA (/Actb)
PMN Mo T B
h
Ratio (%)
Late Apoptosis
Early Apoptosis
ABT-263 (μM)
0 0.1 0.3 1 0 0.1 0.3 1 0 0.1 0.3 1 3 0 0.1 0.3 1 3
Young
Senescent
WT
DKO
MEF
MDSC
i
Before treatment (d0)
Post-treatment (d7)
Population of MDSCs (% in CD45+ cells)
Ctrl
ABT
Mo
PMN

Supplementary Figure 1 MDSCs do not exhibit senescence-associated phenotypes. (a) Cell-cycle analysis of 4',6-diamidino-2-phenylindole (DAPI)-stained cells by flow cytometry; Mo-MDSCs and PMN-MDSCs were harvested from bone marrows (left graphs), spleens (middle graphs), and tumours (right graphs) of SCT-bearing wild-type (WT; black bars) and p16/p21-DKO (DKO; white bars) mice. (b) *In vivo* 5-ethynyl-2′-deoxyuridine (EdU) incorporation assay. Purified splenic PMN-MDSCs, Mo-MDSCs, and intratumor Mo-MDSCs were stained with carboxyfluoroscein diacetate succinimidyl ester (CFSE) and injected intravenously into SCT-bearing mice. Representative flow cytometry plots of recipient Mo-MDSCs ($CSFE^-CD11b^+Gr1^{int}$; left upper graph), PMN-MDSCs ($CSFE^-CD11b^+Gr1^{hi}$; left lower graph), and transferred $CFSE^+$ cells harvested from spleen in WT (middle graphs) and p16/p21-DKO (right graphs) show ratio of EdU incorporation into injected cells during 3 days of exposure to EdU drinking water. (c) *In vitro* CFSE dilution assay by flow cytometry. Purified splenic PMN-MDSCs, Mo-MDSCs were cultured in 20 ng/ml GM-CSF-containing medium for 3 days. Unstimulated T cells (US) and T cells stimulated with anti-CD3/CD28 were served as negative and positive control, respectively. (d-f) Expression levels of P53-binding protein 1 (53BP1) (d) and γ-histone H2AX (γH2AX) (e) in the indicated cell types were analysed by flow cytometry. Upper panels show representative flow cytometry plots and lower panels show average mean fluorescence intensity MFI (n = 3). Mo-MDSCs (MO), PMN-MDSCs (PMN), $CD11b^-$ cells ($CD11b^-$), T cells (T), and B cells (B) harvested from spleens of SCT-bearing mice. Early (Young) and late (Old) passage mouse embryonic fibroblasts (MEFs) served as controls for cellular senescence. (f) Expression of Lamin B1; lysates were prepared from splenic Mo-MDCSs (Mo), PMN-MDSCs (PMN), T cells (T), and B cells (B) of WT TB mice. Immunoblots of Lamin B1 are shown; β-actin served as a loading control. (g) Expression of *Il6* and *Mmp13* mRNA in in splenic PMN-MDSCs (PMN), Mo-MDSCs (Mo), T cells, and B cells from SCT-bearing mice (n = 3), as determined by qRT-PCR. (h) Sensitivity of BM-Mo-MDSCs (see Supplementary Fig. 8) from WT and DKO mice to a senolytic drug (ABT-263). Cells were treated with ABT-263 (0, 0.1, 0.3, 1, or 3 μM) for 72 h and subjected to flow cytometric analysis following annexin V and 7-AAD staining; % frequencies of early apoptotic cells (annexin V-positive and 7-AAD-negative) and late apoptotic cells (annexin V-positive and 7-AAD-positive) are shown. Early passage (Young) and doxorubicin-induced senescent (Senescent) MEFs served as negative and positive controls for ABT-263-induced apoptosis, respectively. (i) Sensitivity of MDSCs in mice to ABT-263. Populations of peripheral blood Mo-MDSCs (MO) and PMN-MDSCs (PMN) in $CD45^+$ cells from mice treated with control vehicle (Ctrl) or 50 mg/kg/d ABT-263 (ABT) once daily for 7 days starting on day 21 post-SCT inoculation are shown. Samples from mice before treatment served as a control (n =7). Data are presented as means ± SEM. The statistical significance was determined by Student's t test; ***$P < 0.001$.

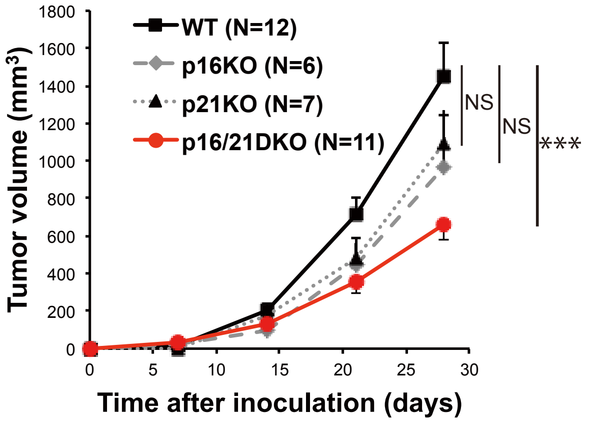

Supplementary Figure 2 Tumour Growth is inhibited in p16/p21-DKO mice but not in p16KO or p21KO mice. Tumour size in SCT-injected WT, p16KO, p21KO, and p16/p21-DKO mice. Data of WT and p16/p21-DKO are also shown in Figure 2a. Data are represented as the means ± SEM. The statistical significance was determined by Student's t test; ***P < 0.001; NS, not significant.

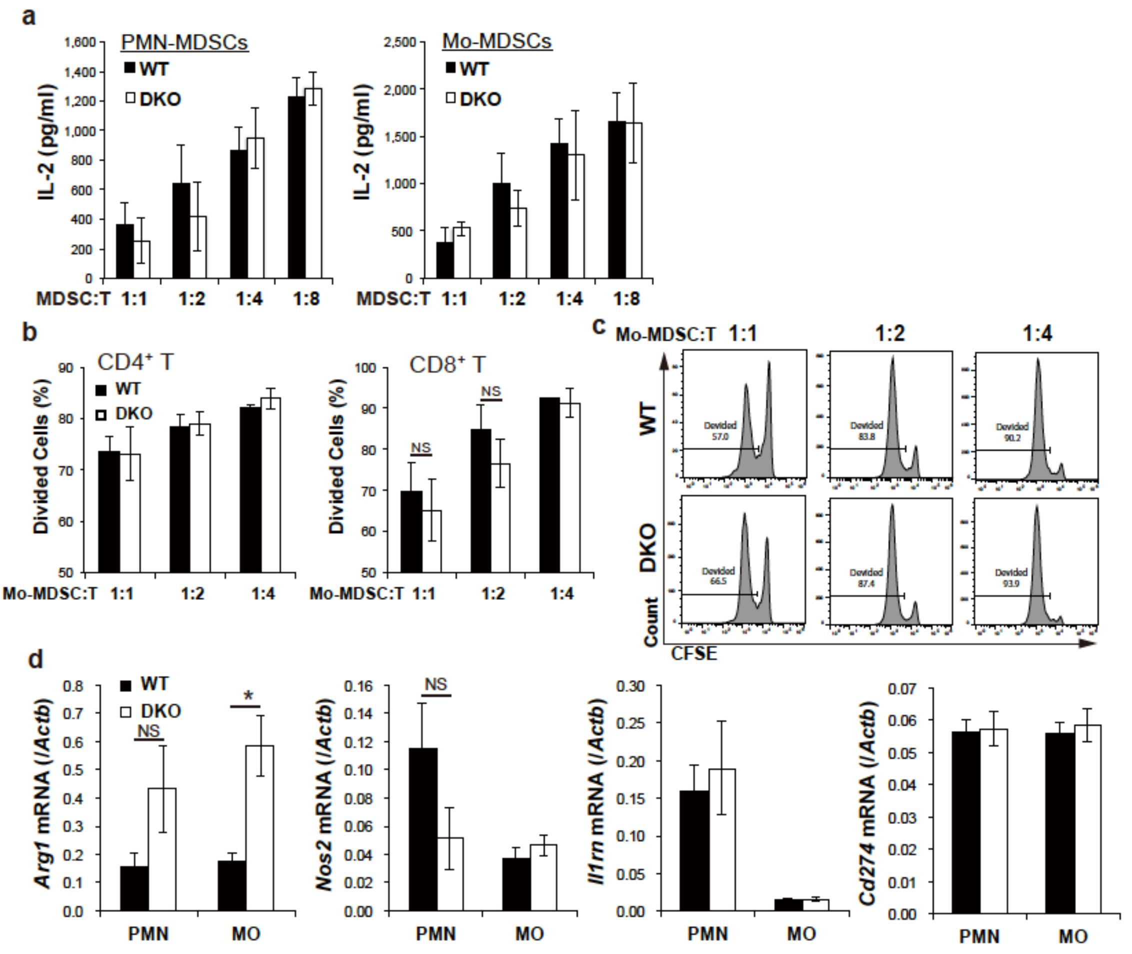

Supplementary Figure 3 There was no significant difference in immunosuppressive ability between MDSCs from WT and those from p16/p21-DKO mice. (a) Antigen-dependent T cell activation *in vitro*; in the presence of OVA-peptide, RF33.70 (OVA-specific T hybridoma) and DC2.4 (dendritic cell line) cells were cultured with purified PMN- (left) and Mo-MDSCs (right) from TB WT or p16/p21-DKO mice at indicated ratios. The level of IL-2 in the culture supernatant, measured by enzyme-linked immunosorbent assay, was used as an indicator of T cell activation. Data represent mean IL-2 concentration (n = 4). (b and c) Cell division analysis of CFSE-stained splenic T cells activated with anti-CD3- and anti-CD28-coated beads in the presence of purified splenic (b) and intratumoural (c) Mo-MDSCs from WT or p16/p21-DKO mice (n = 4). Left and right graphs show mean populations of divided $CD4^+$ and $CD8^+$ cells, respectively. (d) Expression of *Arg1*, *Nos2*, *Il1rn* (IL1R antagonist), and *Cd274* (PD-1L) mRNA in PMN- and Mo-MDSCs from WT or p16/p21-DKO mice (n = 5–6), as determined by qRT-PCR. Data are represented as the means ± SEM. The statistical significance was determined by Student's t test; *P < 0.05; NS, not significant.

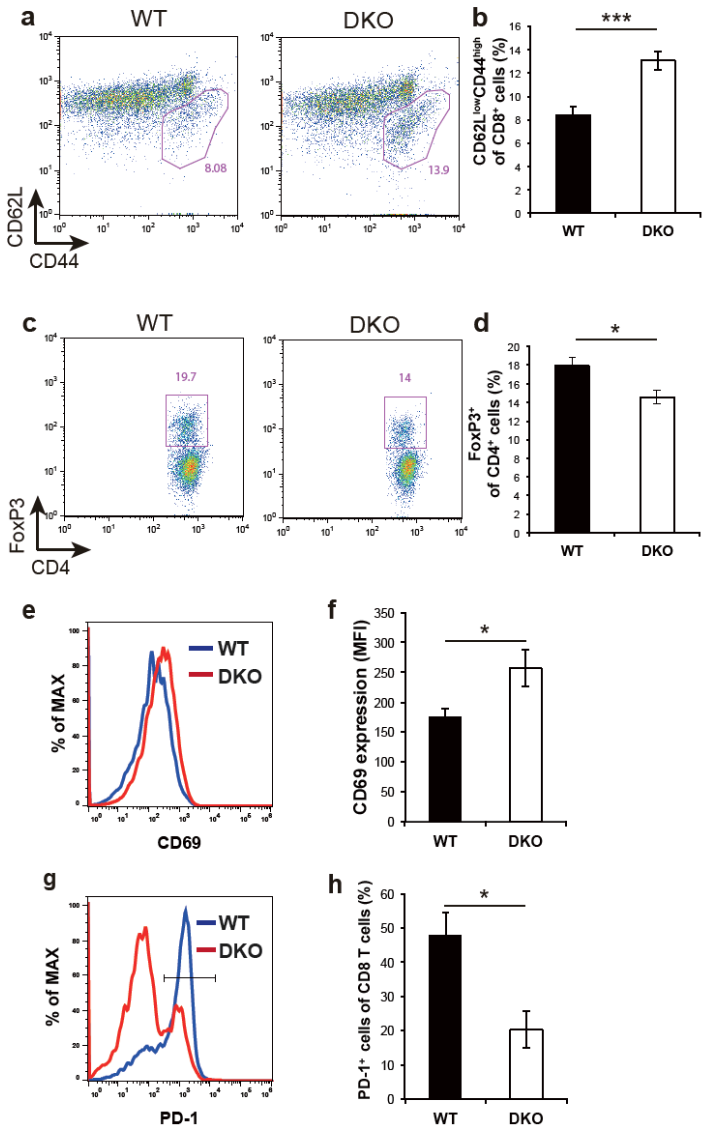

Supplementary Figure 4 Decreased number of intratumoral Mo-MDSCs leads to attenuation of local immunosuppression. (a) Representative flow cytometry plots of CD8^{+}-gated cells in draining lymph nodes of WT and p16/p21-DKO mice 3 weeks after SCT cell injection. Cells were labelled with CD8, CD44, and CD62L antibodies. (b) Mean populations of CD44^{high}CD62^{low} cells in the CD8^{+} gated cells shown in (a) (n = 6); (c) Representative flow cytometry plots of CD3^{+}CD4^{+}-gated cells in draining lymph nodes from WT and p16/p21-DKO mice 3 weeks after SCT cell injection. Cells were labelled with CD3, CD4, and FoxP3 antibodies. (d) Mean number of FoxP3^{+} cells in the CD4^{+} population shown in (c) (n = 6). (e and f) Expression of CD69 in CD8^{+}-gated cells in intratumoural cells. Representative histogram (e) and average of the MFI (f) (n = 4). (g and h) Population of PD-1^{+} cells in CD8^{+}-gated cells in intratumoural cells. Representative histogram (g) and mean (h) (n = 4); data are represented as means ± SEM. The statistical significance was determined by Student's t test; $^{*}P < 0.05$ and $^{***}P < 0.001$.

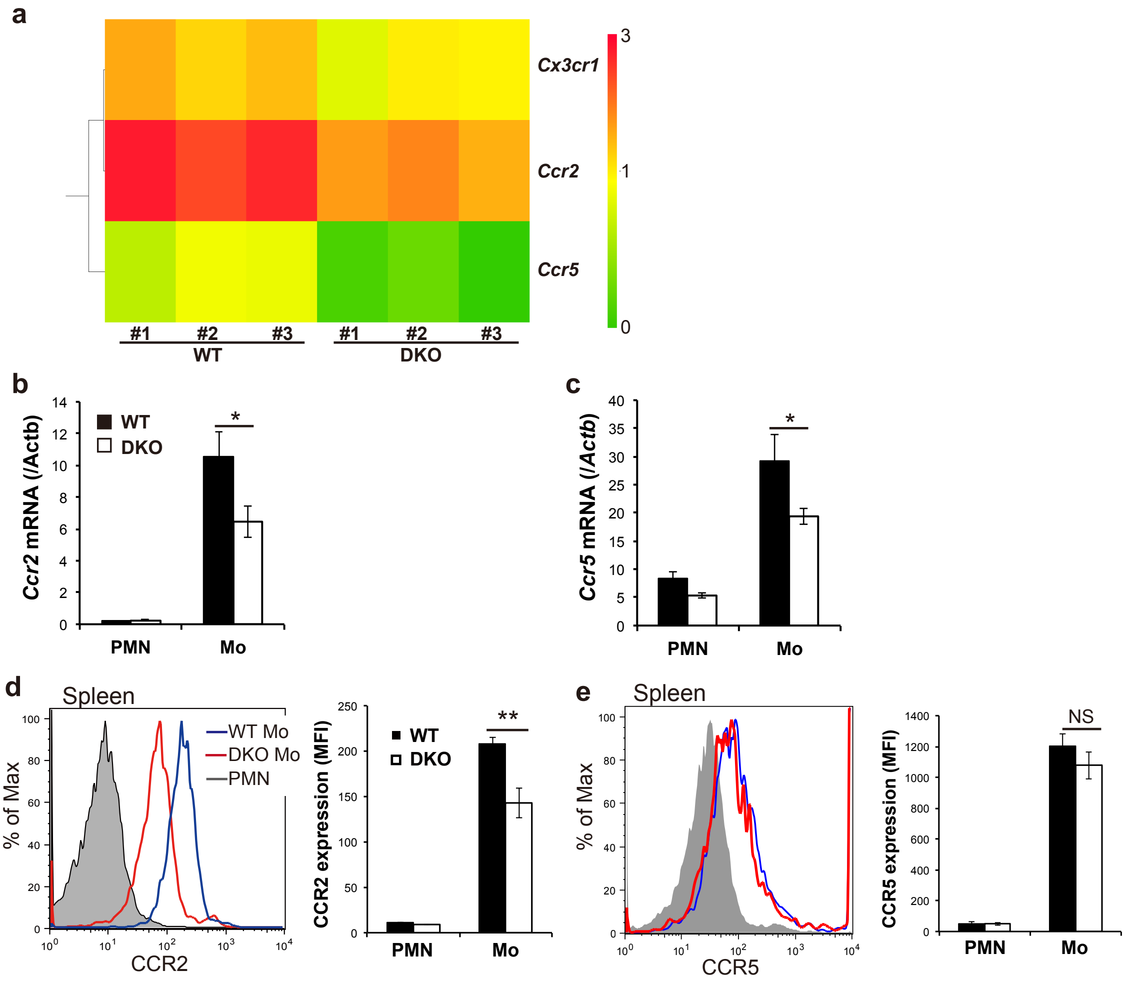

Supplementary Figure 5 Difference in CCR2 and CCR5 expression between WT and p16/p21-DKO Mo-MDSCs. (a) List of genes selected in Figure 3a; mosaic tile representation of each gene is also shown with intensity gradients indicating the mean value of the expression level (log2 ratio). (b and c) Expression of *Ccr2* (b) and *Ccr5* (c) mRNA was measured by qRT-PCR. Representative flow cytometry histograms showing CCR2 (d) and CCR5 (e) expression in PMN- and Mo-MDSCs in spleens from WT and p16/p21-DKO mice 3 weeks after SCT cell injection (n = 5). Graphs are presented as the mean ± SEM. The statistical significance was determined by Student's t test; *P < 0.05 and **P < 0.01; NS, not significant.

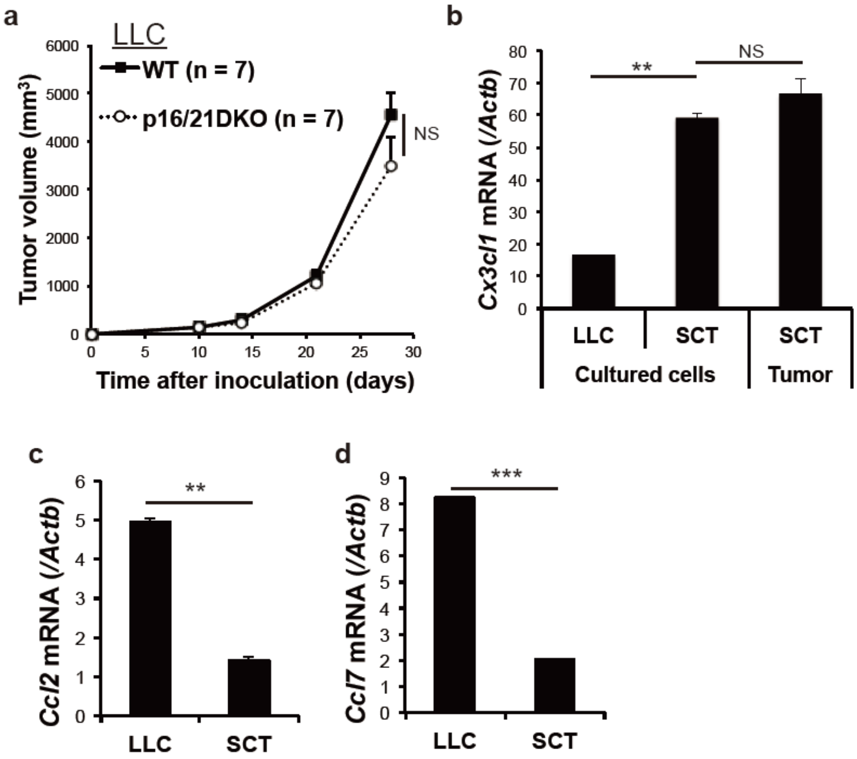

Supplementary Figure 6 Cancer specificity for p16/p21-dependent tumour promotion and CX3CL1 expression in tumour cells. (a) Growth curves of LLC in WT and p16/p21-DKO mice. (b) *Cx3cl1* mRNA expression in the LLC, SCT, and tumour derived from SCT inoculation *in vivo*, as determined by qRT-PCR. (c,d) mRNA level of *Ccl2* (c) and *Ccl7* (d) in LLC and SCT were measured by qRT-PCR. Graphs show the mean ± SEM. The statistical significance was determined by Student's t test; $^{**}P < 0.01$ and $^{***}P < 0.001$; NS, not significant.

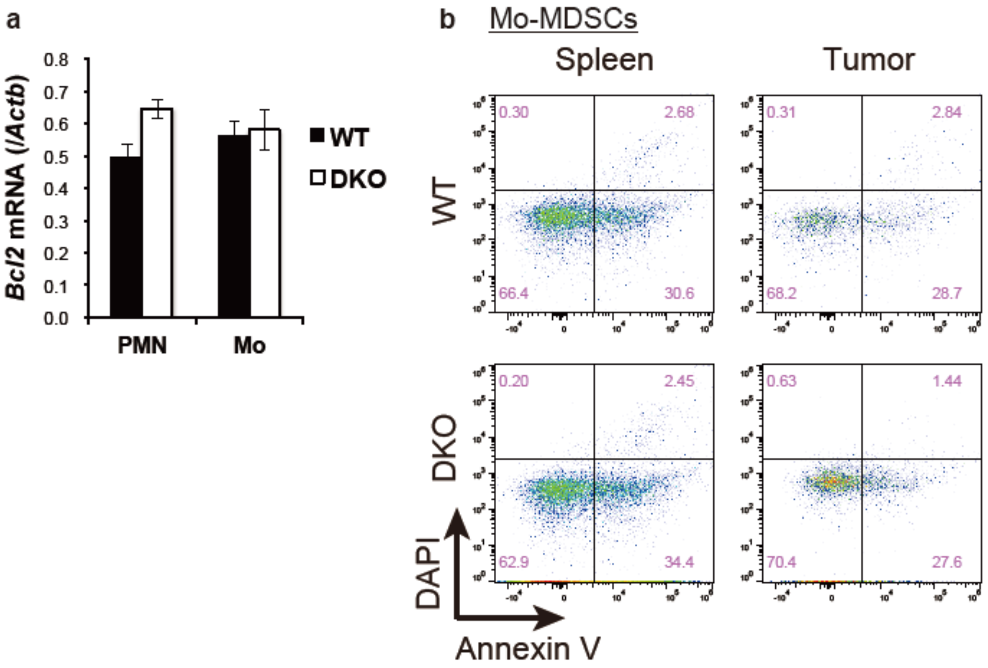

Supplementary Figure 7 Apoptosis in MDSCs. (a) Expression of *Bcl2* mRNA was measured by qRT-PCR. (b) Representative flow cytometry plots of splenic (left) and intratumoral (right) Mo-MDSCs ($CD11b^{+}Ly6C^{hi}Ly6G^{-}$) following annexin V and DAPI staining. Graphs show the mean ± SEM.

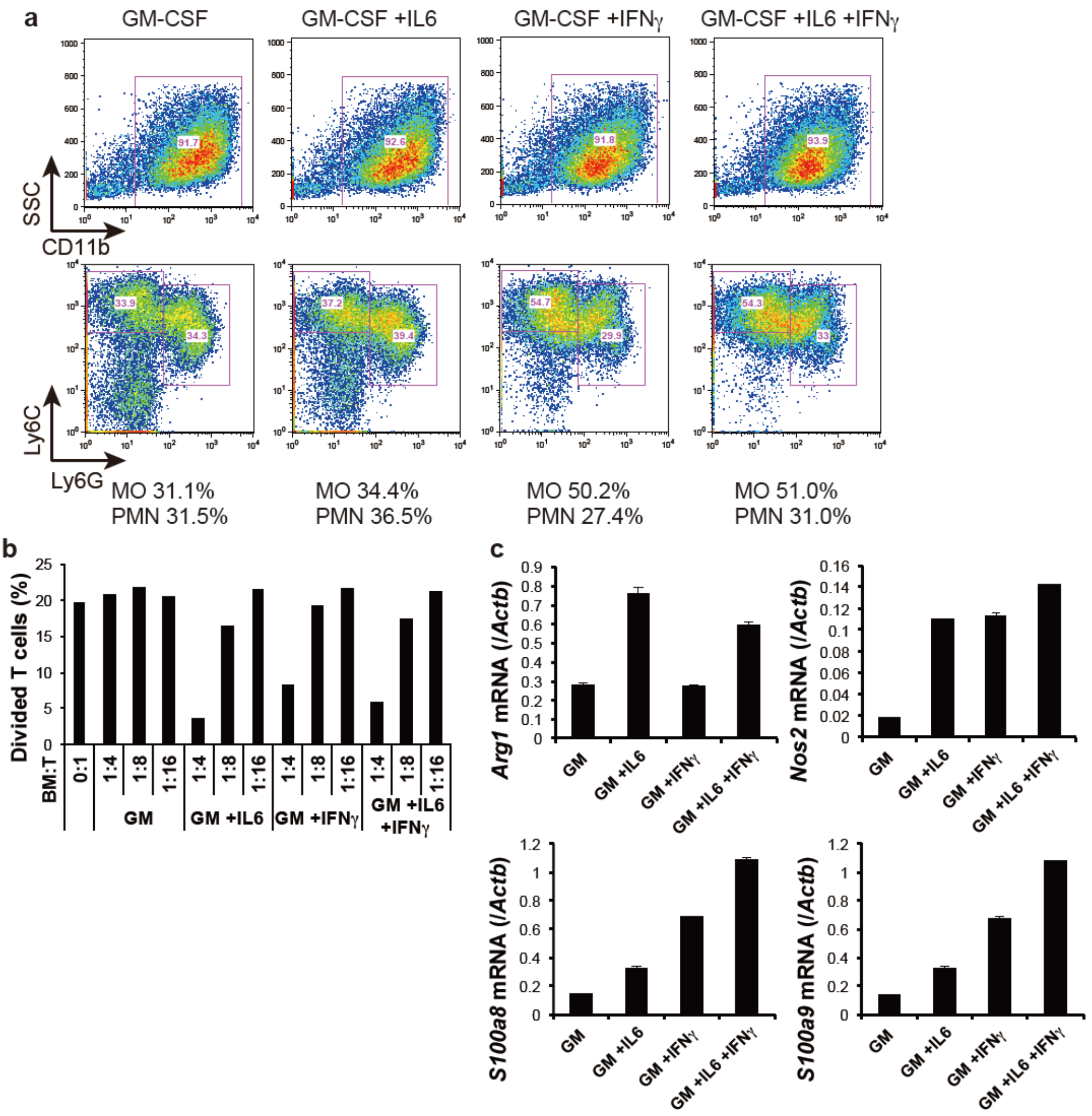

Supplementary Figure 8 IFN-γ enhanced differentiation of Mo-MDSCs from BM cells. (a) Representative flow cytometry plots of BM cells under the indicated culture conditions; cells were labelled with CD11b, Ly6C, and Ly6G antibodies. $CD11b^+$ cells (upper panels) and Mo-MDSC and PMN-MDSC populations among $CD11b^+$ cells (lower panels) were detected. Percentages of Mo-MDSC and PMN-MDSC populations within the total number of cells are indicated. (b) Cell division analysis of CFSE-stained splenic T cells activated with anti-CD3- and anti-CD28-coated beads in the presence of BM-derived cells under indicated culture conditions and BM cell:T cell ratios (c) *Arg1*, *Nos2*, *S100a8*, and *S100a9* mRNA expression in BM-derived cells under the indicated culture conditions was determined by qRT-PCR. Data are representative of three biological replicates. Graphs show the means ± SEM. Data are representative of three independent experiments.

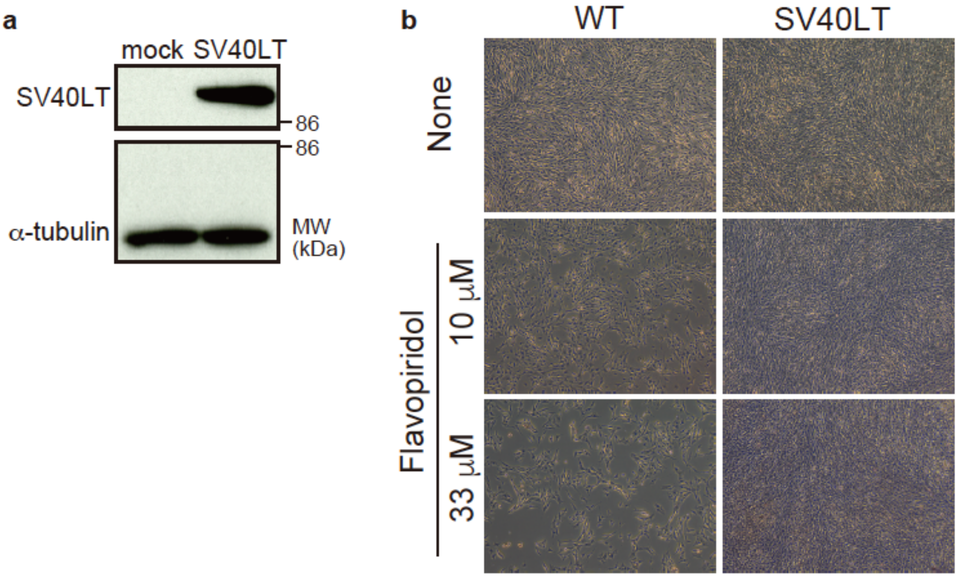

Supplementary Figure 9 SV40LT-expressing SCT cells are resistant to flavopiridol. (a) Expression of SV40 large T antigen (SV40LT); lysates were prepared from mock- and SV40LT-transduced SCT cells. Immunoblots of SV40LT are shown; α-tubulin served as a loading control. (b) Representative images of SCTs and SV40LT-expressing SCTs treated with flavopiridol (33 or 10 μM) or DMSO control (None).

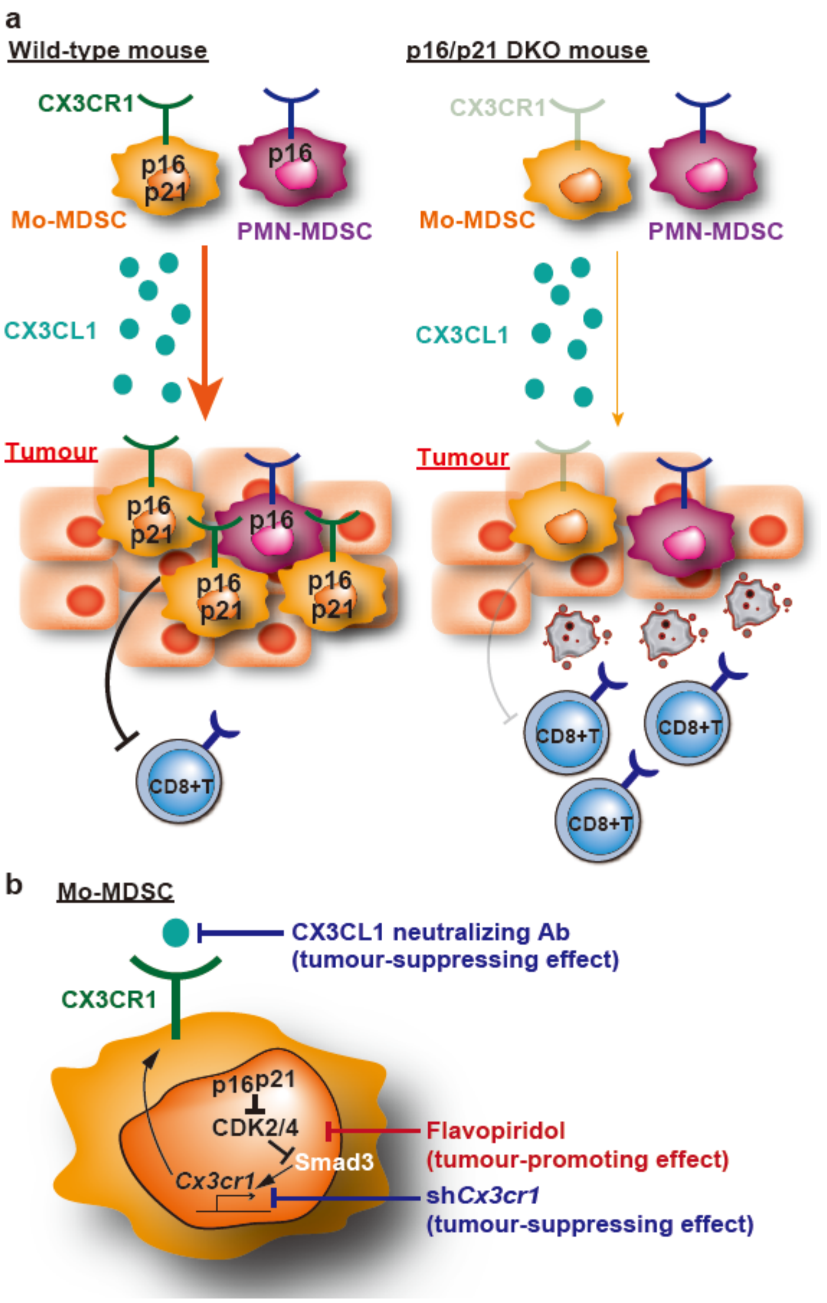

Supplementary Figure 10 Tumour-promoting mechanism of p16^{Ink4}a and p21$^{Cip1/Waf1}$ in Mo-MDSCs. (a) Mo-MDSCs express both p16^{Ink4a} and p21$^{Cip1/Waf1}$, and migrate into CX3CL1-expressing tumours. However, loss of p16/p21 decreases CX3CR1-mediated infiltration of Mo-MDSCs into the tumour, resulting in attenuation of immunosuppressive ability *in vivo*. (b) Molecular mechanism of p16/p21-mediated CX3CR1 expression in Mo-MDSCs and experimentally demonstrated effects of inhibition of the CX3CL1/CX3CR1 axis and CDK on tumour progression.
