# Supplementary material for: p16Ink4a and p21Cip1/Waf1 promote tumour growth by enhancing myeloid-derived suppressor cells chemotaxis
Source: Nat Commun. 2017 Dec 12;8:2050. doi: 10.1038/s41467-017-02281-x (PMC5727112; doi:10.1038/s41467-017-02281-x)
Supplement: Supplementary file 3 — Description of Additional Supplementary Files [file 41467_2017_2281_MOESM3_ESM.pdf]

Description of Additional Supplementary Files

File Name: Supplementary Data 1
Description: **The expression level of angiogenesis-inducing factors.** The table indicates the expression of proangiogenic genes in Mo-MDSCs harvested from spleens of WT and p16/p21-DKO mice 3 weeks after tumour inoculation. Data was analyzed from RNA-sequencing (related to Fig. 3a, Supplementary Fig. 5a, Supplementary Data 2).

File Name: Supplementary Data 2
Description: **The expression level of EMT-inducing factors.** The table indicates the expression of epithelial-mesenchymal transition (EMT)-related genes in Mo-MDSCs harvested from spleens of WT and p16/p21-DKO mice 3 weeks after tumour inoculation. Data was analyzed from RNA-sequencing (related to Fig. 3a, Supplementary Fig. 5a, Supplementary Data 1).

File Name: Supplementary Data 3
Description: **List of Oligonucleotides.** Oligonucleotides primers used in the study.

File Name: Supplementary Data 4
Description: **List of Antibodies.** Antibodies used in the study.
